# Application of an Array of Metal-Oxide Semiconductor Gas Sensors in an Assistant Personal Robot for Early Gas Leak Detection

**DOI:** 10.3390/s19091957

**Published:** 2019-04-26

**Authors:** Jordi Palacín, David Martínez, Eduard Clotet, Tomàs Pallejà, Javier Burgués, Jordi Fonollosa, Antonio Pardo, Santiago Marco

**Affiliations:** 1 Universitat de Lleida, Jaume II, 69, 25001 Lleida, Spain; palacin@diei.udl.cat (J.P.); david.martinez@diei.udl.cat (D.M.); eclotet@diei.udl.cat (E.C.); tpalleja@diei.udl.cat (T.P.); 2 Department of Electronics and Biomedical Engineering, Universitat de Barcelona, Marti i Franqués 1, 08028 Barcelona, Spain; apardo@el.ub.edu (A.P.); santiago.marco@ub.edu (S.M.); 3 Signal and Information Processing for Sensing Systems, Institute for Bioengineering of Catalonia (IBEC), The Barcelona Institute of Science and Technology, Baldiri Reixac 10–12, 08028 Barcelona, Spain; 4 Centre de Recerca en Enginyeria Biomèdica, CREB. Enginyeria de Sistemes, Automàtica i Informàtica Industrial, Universitat Politècnica de Catalunya, 08028 Barcelona, Spain; jordi.fonollosa.m@upc.edu; 5 Biomedical Research Networking Center in Bioengineering, Biomaterials and Nanomedicine CIBER-BBN, 28029 Madrid, Spain; 6 Institut de Recerca Pediàtrica Hospital Sant Joan de Déu, 08950 Esplugues de Llobregat, Spain

**Keywords:** metal-oxide semiconductor, gas sensor, gas leak detection, assistant personal robot, mobile robot

## Abstract

This paper proposes the application of a low-cost gas sensor array in an assistant personal robot (APR) in order to extend the capabilities of the mobile robot as an early gas leak detector for safety purposes. The gas sensor array is composed of 16 low-cost metal-oxide (MOX) gas sensors, which are continuously in operation. The mobile robot was modified to keep the gas sensor array always switched on, even in the case of battery recharge. The gas sensor array provides 16 individual gas measurements and one output that is a cumulative summary of all measurements, used as an overall indicator of a gas concentration change. The results of preliminary experiments were used to train a partial least squares discriminant analysis (PLS-DA) classifier with air, ethanol, and acetone as output classes. Then, the mobile robot gas leak detection capabilities were experimentally evaluated in a public facility, by forcing the evaporation of (1) ethanol, (2) acetone, and (3) ethanol and acetone at different locations. The positive results obtained in different operation conditions over the course of one month confirmed the early detection capabilities of the proposed mobile system. For example, the APR was able to detect a gas leak produced inside a closed room from the external corridor due to small leakages under the door induced by the forced ventilation system of the building.

## 1. Introduction

The combination of mobile robots and environmental sensors enables the automatic supervision of environmental parameters in large areas, for example, to reduce energy consumption or guarantee human comfort. For example, Martínez et al. [1] described a mobile robot used in an ambient intelligent application to detect uncomfortable temperature profiles. Similarly, Palacín et al. [2] showed the automatic supervision of temperature, humidity, and luminance to evaluate human comfort in largely frequented areas and to optimize the energy spent in heating, ventilation, and air conditioning (HVAC) and in illumination. Jin et al. [3] measured the performance of a ventilation system for room air renewal by means of a mobile robot equipped with carbon dioxide (CO_2_) and volatile organic compound (VOC) sensors and a stationary sensor network, showing that the robot can provide more accurate estimates of the air quality. Mobile robots equipped with gas detectors were also used in outdoor applications for pollution monitoring and source localization in public areas [4], surveillance of industrial facilities producing harmful gases [5], and monitoring of landfill sites [6]. More recently, gas monitoring outdoors was also addressed using unmanned aerial vehicles (UAVs), although with more size and power constraint limitations. For example, Rossi et al. [7] proposed a fully autonomous UAV gas sensing system based on the use of metal-oxide (MOX) sensors with an autonomy of 30 minutes, capable of providing wireless real-time feedback. Gallego et al. [8] proposed an optimization of the speed of the UAV and of the power consumption based on the gas sampling frequency. Additionally, Rossi et al. [9] proposed a battery-powered, lightweight, and compact gas sensing board based on the use of two MOX sensors, which was suitable for any type of mobile carrier such as UAVs or wheeled robots.

The field of mobile robotic olfaction (MRO) traditionally considers two main tasks: gas source localization (GSL) and gas distribution mapping (GDM). GSL consists of finding the source of a released chemical, while GDM aims at building a map of the distribution or spread of the chemical in the environment. GSL algorithms [7,8,9,10,11,12,13,14,15] can be divided into reactive plume tracking, plume modeling, or map-based approaches. They usually assume that a single gas source is present in the environment. GDM strategies [16] typically require that the robot fully explores the environment, and some of the works considered multiple gas sources [17,18,19,20,21]. Loutfi et al. [17] obtained classification rates higher than 85% in experiments where two odor sources were placed in different corridors to facilitate the discrimination task. Monroy et al. [21] studied the impact of the speed of the robot in the discrimination of two chemicals using a mobile robot equipped with an e-nose. They found that the classification accuracy can degrade up to 30% when the motion speed of the data used for training highly differs from that of the testing. The same authors proposed a method to create time-variant maps using obstacle information [19]. One of the main limitations of previous indoor works was the overly simplistic scenarios in terms of distance between the robot and the source. In most cases, the robot started the exploration in the same room where the source was located, only a few meters away. There are few exceptions to this. For example, Burgués et al. [19] used a gas-sensitive UAV to explore an indoor area of 160 m^2^.

The use of MOX gas sensors in mobile robots has the advantage of its lower cost and weight with respect to other technologies. On the other hand, MOX sensors have well-known limitations such as their limit of detection [22], power consumption [23], and their lack of selectivity and cross-sensitivity to environmental factors such as temperature and humidity [24]. By combining multiple MOX sensors with different operating temperatures, the selectivity of the system can be increased. Arrays of MOX sensors, commonly known as e-noses, are widely used for gas sensing in ambient monitoring [25] and gas distribution mapping [18,26,27]. For example, arrays of six [18,27] and 10 [21] MOX sensors were used in a mobile robot in order to create indoor odor maps in presence of multiple sources. Finally, Hernández et al. [18] combined a low-cost MOX array for chemical identification and a photo-ionization detector (PID) to obtain fast and calibrated measurements of the concentration of the gas detected.

This paper proposes the application of an array of MOX gas sensors as a cost-affordable sensor embedded in an assistant personal robot (APR) initially designed to provide telepresence [28], transport of small objects, and other assistive services [29]. The final goal is the inclusion of gas sensing capabilities in this type of mobile robot in order for it to operate as an early gas leak detection system. However, the mobile robot will also have application as a specific tele-controlled or autonomous exploratory device for the detection and identification of gas leaks in hazardous conditions. The gas sensing capabilities are based on a custom array composed of 16 low-cost MOX gas sensors, based on the definition of an overall gas detector procedure, and on the application of a multivariate classification model (partial least squares discriminant analysis; PLS-DA) for gas leak identification. 

The main contribution of this paper is the validation of the application of an array of MOX gas sensors in a mobile robot for early gas leak detection in a real case scenario. The novelty of this contribution lies in the number of MOX sensors used, on the low concentrations of the target gas, on the presence of chemical interferences, and on the dimensions (15 m × 40 m) and complexity of the testing environment. These conditions represent an improvement over similar validations performed in simulated scenarios [13] or in smaller areas [19] containing a single gas source [26] and artificially induced unidirectional airflows [30]. The experimental arena is one floor of a university building, in which a hypothetic gas leak can potentially spread very fast and affect many people. In this scenario, the APR performing a routine patrol [1,2] must detect, as early as possible, gas leaking from a gas source located far from the initial position of the robot or even in a closed room. 

## 2. Methods

### 2.1. Assistant Personal Robot

The mobile robot used in this paper is a second-generation APR prototype [28] (APR-02) which includes a holonomic motion system [31] and an improved suspension designed to absorb the vibrations generated by the displacement of the omnidirectional wheels [32].

**Figure 1:**
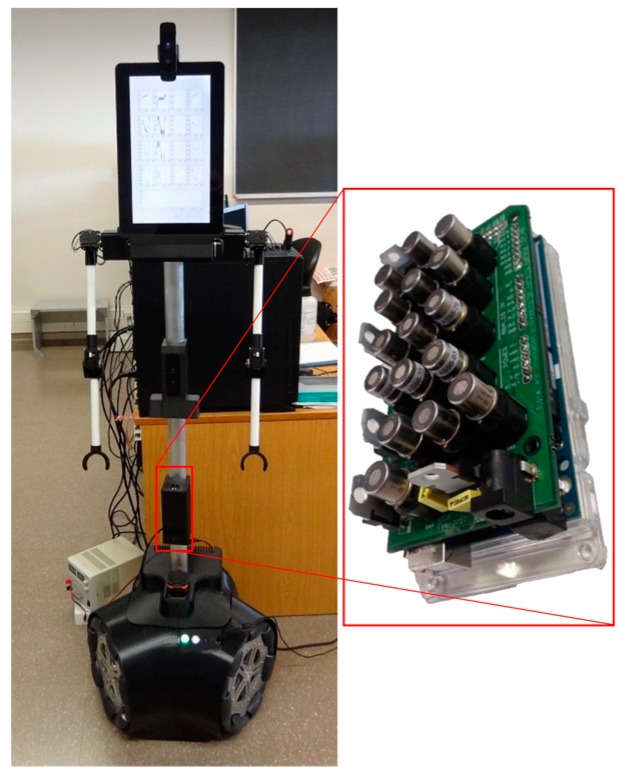
Image of the assistant personal robot (APR-02) and detail of the array of metal-oxide (MOX) gas sensors used for early gas leakage detection.

The APR-02 (Figure 1) includes a central processing unit based on a 4.2 GHz Intel(R) Core (TM) I7-7700K processor, 16 GB of Double Data Rate 4 (DDR4) random-access memory (RAM), and a 250 GB Western Digital PCIE NVMe (non-volatile memory express) solid-state drive (SSD) disc. The connectivity of the central processing unit with all embedded sensors is based on Universal Serial Bus (USB) connectivity, registering the devices as active USB-COM drivers. All sensors are accessed using standard functions for the RS232 serial port which are managed by an initialization agent that shares the information of the sensors between the different programs, functions, and agents that define a particular application of the APR [33].

The APR-02 uses a two-dimensional (2D) light detection and ranging (LIDAR; UTM-30LX by Hokuyo) as a main sensor for SLAM [34,35]. The mapping and autonomous navigation capabilities are based on the application of the iterative closest point algorithm (ICP) [36] which, depending on the configured operation, uses the scan data gathered with the LIDAR sensor in order to estimate the position of the robot relative to a previous scan or relative to a reference map, either creating and/or updating the current map with the information of the current scans. Finally, the A* [37,38] algorithm is used to compute an optimal path between the initial robot position and its target destination according on the information available in the current active map.

### 2.2. Gas Sensor Array

Figure 1 shows details of the gas sensing array used in the APR-02. According to previous experiences in gas leakage detection experiments with mobile robots [39,40], the gas sensing array is placed in front of the mobile robot at a height of 500 mm. The gas sensing system is a heterogeneous array of 16 MOX sensors (e-nose) which is an evolution of the array of eight MOX sensors proposed to detect two gas sources in a wind tunnel by Fonollosa et al. [41]; however, in this paper, the gas sensor array will be used to sample in open conditions. The gas sensing array is complemented with a ppbRAE 3000 PID (RAE Systems) for validation purposes, providing accurate gas concentration information. The gas sensing array integrates 16 MOX gas sensors (4× FIGARO TGS 2600, 4× FIGARO TGS 2602, 4× FIGARO TGS 2611, 4× FIGARO TGS 2620) in a 105.06 × 57.90 mm printed circuit board (PCB) that contains the interface and conditioning electronics. The MOX sensors are always turned on in order to operate them isothermally, as recommended by the manufacturer.

Similarly to Rossi et al. [9], the specific power applied to each heating resistor is controlled by a pulse width modulation (PWM) signal to change the temperature of the sensing layer. This PWM operates at 500 Hz and the heating resistors are powered with 6.5 V. Sensors of the same model are powered with different duty cycles (Table 1), which results in different operating temperatures and different sensitivity profiles [42]. When sensors of the same type are operated at different temperatures, the multivariate sensor responses can be processed to increase the selectivity [43]. The gas sensing array includes an Arduino Mega microcontroller which controls the power applied to the heating resistors, reads the sensor signals, and provides external USB serial connectivity. The readout circuit is based on a voltage divider using a load resistor (R_L_) of 10 kΩ, which is connected to a reference voltage (V_REF_) of 3.3 V, and the sensing resistor (R_S_), which is connected to the ground. The sensor voltage is measured at 10 Hz with a 10-bit analog-to-digital converter (ADC). Hence, the value of the sensing resistor (R_S_) can be analytically deduced from the digital value provided by the ADC.

**Figure 2:**
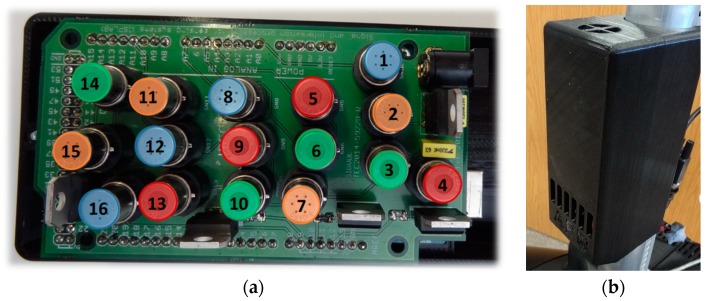
(**a**) Distribution of the MOX sensors included in the electronic board: FIGARO TGS 2600 (blue: 1, 8, 12, 16), FIGARO TGS 2602 (orange: 2, 7, 11, 15), FIGARO TGS 2611 (green: 3, 6, 10, 14), FIGARO TGS 2620 (red: 4, 5, 9, 13). (**b**) Enclosure of the sensor array integrated with the APR (160 × 60 × 50 mm).

Figure 2 shows the sensor board with the spatial distribution of the 16 MOX sensors and the sensor enclosure designed and implemented in black polylactic acid (PLA). The sensor case includes entry air holes in the bottom and a small ventilation hole in the top. The upper hole includes an internal extractor fan (25 × 25 × 8 mm, 3.2 m^3^/h, 500 mW, 12 V of direct current (DC)) as a way to force a slightly but constant air circulation of air inside the case of the device. The fan was powered at 6 V in order to obtain an optimal air circulation inside the device with a minimum cooling effect in the sensors. 

The mobile robot has a power management procedure which is continuously measuring the power drained from the batteries and continuously providing an estimate of the remaining battery capacity in order to generate a battery recharge warning. The maximum power consumption of the gas sensor array is approximately 1.0 A (powered at 12 V of DC). This continuous power consumption represents approximately 2.7% of the current onboard batteries of the APR-02 prototype. In this paper, the continuous powering of the gas sensor array is guaranteed by increasing the threshold level of the battery warning in order to have enough time to return to the recharging station. Similarly, the recharging system keeps powering the gas sensor array even if the mobile robot is completely turned off. The gas sensor array was operated continuously during several months without any remarkable incidence. However, future evolutions must include procedures to reduce the power consumption while preserving the accuracy of the measurements. For example, Rossi et al. [44] proposed a strategy of sampling and processing which permits reducing the energy consumption by one order of magnitude. Jeličić et al. [45] proposed the use of a pyroelectric infrared sensor to modulate the duty cycle of an MOX gas sensor only when detecting the presence of people. Rossi et al. [46] and Brunelli et al. [47] also proposed the strategy of fixing a reading interval allowing a 20-fold reduction of the power required to perform measurements with an MOX gas sensor.

### 2.3. Odor Delivery System

**Figure 3:**
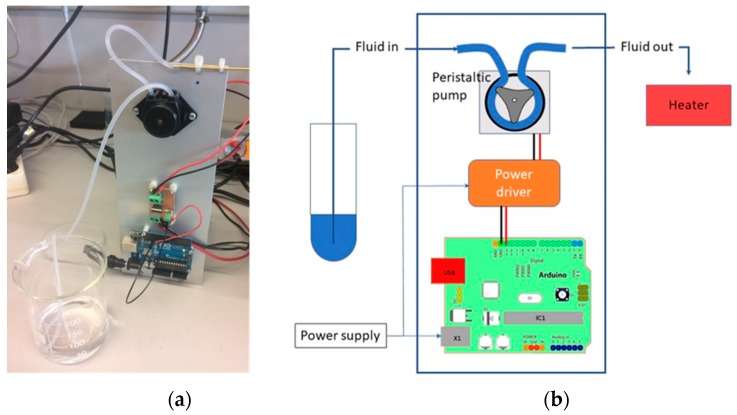
Odor delivery system: (**a**) photo; (**b**) schematic.

Ethanol and acetone were selected to carry out the gas leak experiments as they are commonly used in scientific experimentation with MOX gas sensors [42,47] in open spaces due to their low toxicity. The analytes in liquid form were dosed over a heated surface for its evaporation. Figure 3 shows the evaporation system, based on a peristaltic pump (0.5 mL/min), for accurate liquid-flux control and a 30-W heating resistor as a final evaporator. 

### 2.4. Experimental Area

**Figure 4:**
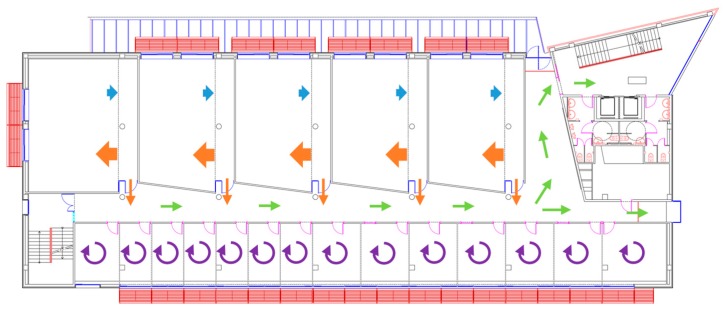
Detail of the experimentation arena and representation of the air circulation.

The experiments of early gas detection with the APR and the onboard matrix of MOX gas sensors were performed on the second floor of the Polytechnic School located in the University of Lleida, Spain. Figure 4 shows a map of this area, which contains a large corridor with one emergency exit door (which is normally closed) at the end and one hall at the beginning. There are also 14 small offices on one side and five laboratories and multiuse rooms on the other side. In this mixed area, a hypothetic gas leak can potentially spread very fast and affect many people. Figure 4 also shows a representation of the global air recirculation originated from the central HVAC system with different arrows. In the laboratories, the conditioned air comes through air vents located in the ceiling. On the one hand, the injection of air originates a slight pressure inside the laboratories that is passively drained with return ducts also located in the ceiling. However, the air is also pushed below closed doors, originating a global air recirculation in the building. Therefore, one of the hypotheses is that a gas leakage originating inside a laboratory will reach the corridor and the hall of the plant, even in the case of a laboratory with a closed door. On the other hand, the HVAC of the offices is based on individual fan coil units (FCU) that do not increase the air pressure and do not contribute to the global air recirculation in the building. The air current profiles described in Figure 4 were measured by Martinez et al. [1].

### 2.5. Measurement Campaigns

The robot equipped with the MOX sensor array and the PID was used to collect data in the scenario described in Section 2.4. The first measurement campaign, performed at the beginning of May 2018, consisted of 11 explorations of the target area, divided into three explorations with clean air conditions, four explorations with an ethanol gas source placed in the middle of the corridor, and four explorations with an acetone gas source in the same location. The experimental protocol consisted of continuously evaporating the selected analyte using the gas source described before and displacing the mobile robot towards the gas flow. The initial position of the robot was around 10 m from the gas source. In each exploration, which lasted approximately four minutes, the robot traversed the corridor (following the green lines depicted in Figure 4) from one end to the other at a speed of 0.13–0.16 m/s without stopping. The HVAC of the building was switched on at all times. This dataset was used for building the multivariate classifier.

The second measurement campaign, performed at the end of June 2018, was used for validation purposes. The robot performed several explorations of the corridor using the same trajectory as in the previous measurement campaign, but we devised four different scenarios. In Scenario I, a single gas source (ethanol or acetone) was placed in the middle of the corridor (same location as in the previous campaign) and the HVAC of the building was switched on. In Scenario II, the gas source of acetone was placed in the same location as before, but the HVAC of the building was switched off to explore the effect of ventilation in gas source localization. In Scenario III, we placed two gas sources (ethanol and acetone) in different locations of the corridor and the HVAC was switched off to avoid premature mixing of the two substances. In Scenario IV, the gas source of ethanol was placed inside one of the rooms adjacent to the corridor and the robot needed to detect the presence of the source from outside the room with the door closed (just from propagation below the door).

### 2.6. PLS-DA Classifier

A PLS-DA classifier [48] was trained to discriminate in real time between three classes (air, ethanol, and acetone) from the raw uncalibrated data gathered from the gas sensing array; the data obtained with the PID were omitted in this training since the response time of both devices was significantly different and could lead to inaccurate classification. PLS-DA is especially suited to deal with multicollinearity, one of the main problems encountered when analyzing gas sensor array data. The number of latent variables (LV) of the model was set to three based on previous experience of the authors. The model was built using all data from the first measurement campaign. The classification model was validated in real time using the data captured in the second measurement campaign. We considered that the gas identification was correct when the classifier output went from air (far from the source) to the correct gas when the robot approached the source. In case both labels (ethanol and acetone) appeared at the classifier output, we considered the identification correct when the correct gas label was displayed longer than the alternative gas label.

## 3. Results and Discussion

This section describes the experimental results and discusses them in the perspective of early gas leak detection.

### 3.1. Signals Acquired in the First Measurement Campaign

**Figure 5:**
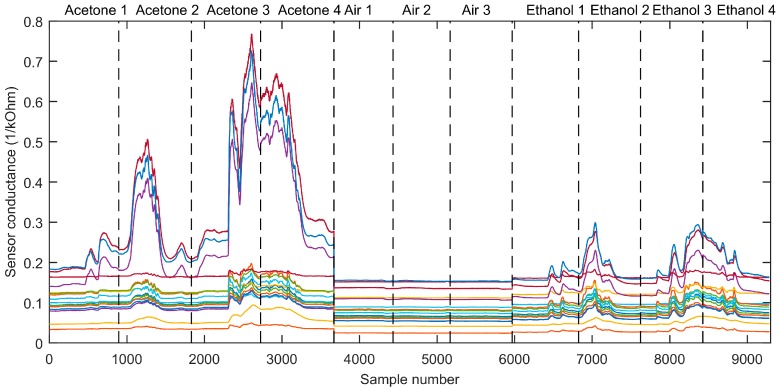
Measured sensor conductance obtained with the 16-element MOX sensor array when the APR traveled through the arena and reached the gas plume for ethanol and acetone.

The main goal of the first measurement campaign was to confirm that the sensors had a limit of detection (LOD) good enough to have a sufficient response to ethanol and acetone in the different scenarios, and also to build a PLS-DA classifier. Figure 5 shows the raw conductance measured from each of the 16 MOX sensors when the mobile robot moved toward a gas source evaporating ethanol or acetone at a flow rate of 0.5 mL/min, or when it explored the area and no gas source was present. The underlying hypothesis was that the different sensor types and the different power applied to sensor heaters of the same type may induce different responses. As a result, the multivariate sensor response will enable the ethanol/acetone discrimination task.

### 3.2. Calibration of the PLS-DA Classifier

**Figure 6:**
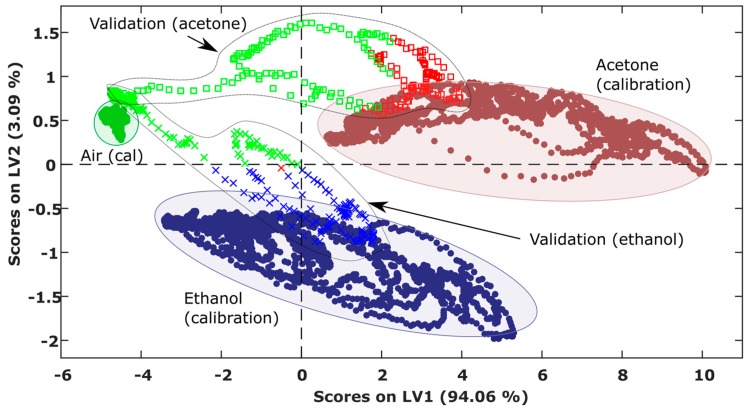
Projection of two validation runs with one gas source (crosses and squares) into the partial least squares discriminant analysis (PLS-DA) score plot of calibration data (filled circles). The validation data are colored according to the label given by the classifier.

Using the raw signals shown in Figure 5, we built a PLS-DA classifier with 3 LVs. The scores of the calibration samples in the latent variable space (Figure 6) indicated good separability between the three classes. To validate the performance of the classifier in unseen data, we projected data from the second measurement campaign (Scenario I) into the score plot obtained from the first campaign and colored the samples according to the classifier output. In the test samples, the beginning of the robot trajectory was classified as clean air (green crosses and squares) because the robot was far from the source, and then the predicted class converged to the true class (blue crosses and red squares) as the robot approached and passed by the source.

### 3.3. Scenario I: One Gas Source and HVAC Turned On

**Figure 7:**
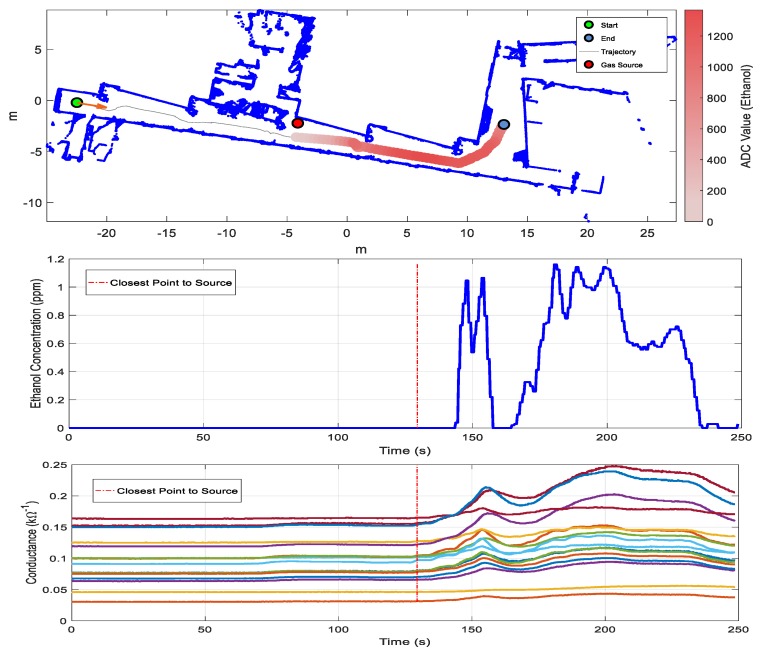
Results of exploration with one gas source of ethanol (Scenario I). From top to bottom: map of the arena (built by the robot) and output of the PLS-DA classifier overlaid on the robot trajectory; concentration measured by the photo-ionization detector (PID); conductance of the matrix of MOX gas sensors; output of the PLS-DA classifier and mean sensor array response (analog-to-digital converter (ADC) value) (for visual clarity, we added a threshold that indicates the mean array response during the commutation from air to ethanol). The red dotted line indicates the point of closest approximation to the gas source.

This sub-section shows the exploration results obtained with the mobile robot and the matrix of MOX gas sensors in the case of a gas leak source located approximately in the middle of the corridor. Figure 7 shows the exploration trajectory followed by the mobile robot, the position of the gas source (red circle), and the information of the mean sensor array response. The cumulative sum of the ADC value was used as an overall indicator of the existence of a variation of conductance caused by a variation in the concentration of volatile substances. A threshold level applied to this cumulative sum was used as a fast detector. In this case, the threshold level was reached just after the robot passed next to the source and the classifier correctly classified the gas as ethanol. The conductance of the matrix of MOX sensors had a similar evolution to the PID signal, which we considered ground truth. The PID registered peak concentrations of approximately 1.1 ppm next to the gas source and along the corridor and the hall of the plant. These low concentrations represent a challenging scenario for MOX sensors, which exhibit LOD values around 1 ppm even with multivariate calibration models in the presence of humidity interference [49]. The mean sensor array response reached its peak value at almost five meters from the source. The main reason for this phenomenon was the vertical distance between the gas source (located at ground level) and the gas sensor (55 cm above the ground), combined with the air currents induced by the HVAC system, causing the plume to be pushed away from the source. This was already observed by Sánchez-Sosa et al. [30] and is later confirmed by results obtained with the HVAC system turned off. 

**Figure 8:**
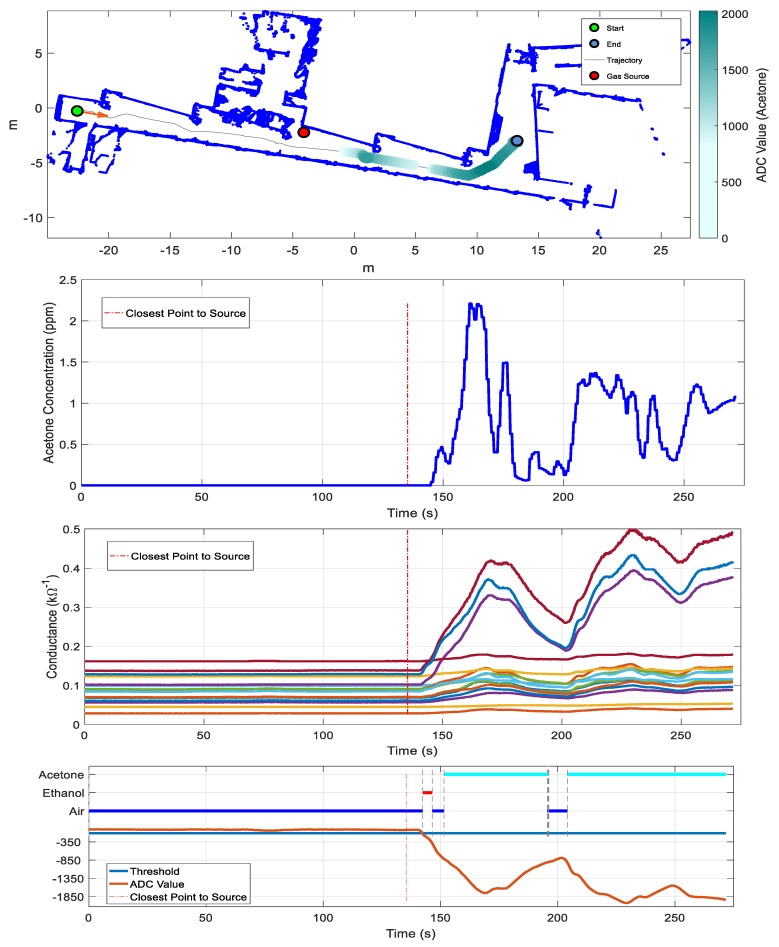
Results of exploration with one gas source of acetone (Scenario I). From top to bottom: map of the arena (built by the robot) and mean sensor array response overlaid on the robot trajectory; concentration measured by the PID; conductance of the matrix of MOX gas sensors; output of the PLS-DA classifier and mean sensor array response (ADC value) (for visual clarity, we added a threshold that indicates the mean array response during the commutation from air to ethanol). The red dotted line indicates the point of closest approximation to the gas source.

Similarly to the previous example of ethanol presentation, we released acetone in the environment. Figure 8 shows the trajectory, the sensor array response, and the output of the classifier. A variation in the conductance of the sensors was observed when the robot passed by the acetone gas source, as in the case of ethanol. Peak concentrations of approximately 2.2 ppm next to the gas source were measured by the PID. The output of the classifier presented some transition time to correctly predict the class of acetone. Results agreed with the hypothesis that there is global air recirculation into the plant that pushes the air from the laboratories to the hall. In fact, the sudden descent of volatile concentration appearing in the trajectory of the mobile robot was probably caused by the arrival of new fresh air that entered into the global recirculating air flow from below the closed door corresponding to the next laboratory in the corridor.

### 3.4. Scenario II: One Gas Source and HVAC Turned Off

**Figure 9:**
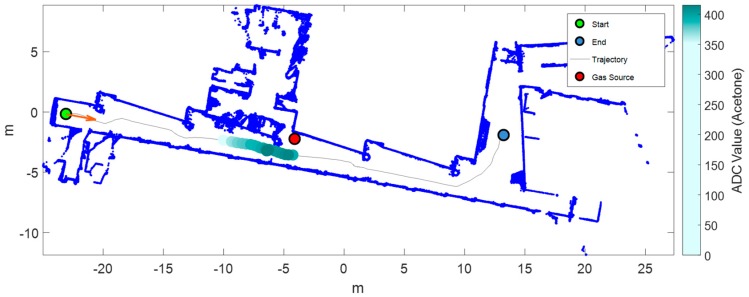
Results of exploration with one gas source of acetone and heating, ventilation, and air conditioning (HVAC) turned off (Scenario II). Map of the arena (built by the robot) and mean sensor array response overlaid on the robot trajectory.

The robot explored the arena with a gas source of acetone in the middle of the corridor with the HVAC switched off. Figure 9 represents the mean response of the sensor array overlaid on the robot trajectory. Compared to the results shown in Figure 8 (same conditions but HVAC turned on), the current map shows less diffusion of the evaporated acetone due to the inexistence of global recirculating air currents in the building. This allows a more precise estimation of the source location using the sensor array.

### 3.5. Scenario III: Two Gas Sources and HVAC Switched Off

**Figure 10:**
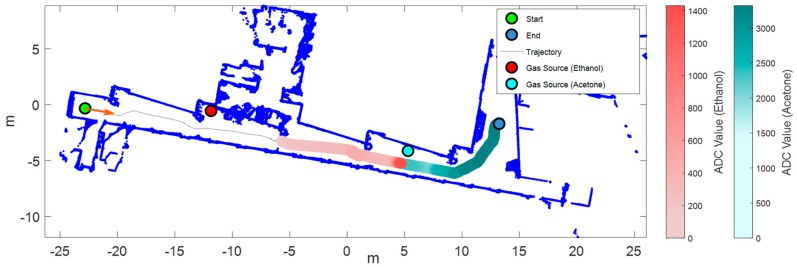

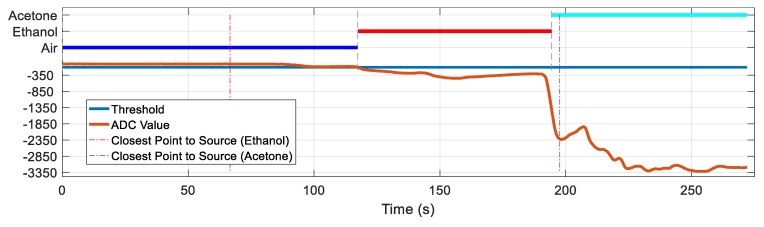
Results of exploration with two gas sources (ethanol and acetone) and HVAC turned off (Scenario III). Top: map of the arena (built by the robot) and mean sensor array response overlaid on the robot trajectory using two colors based on the classifier output (red for ethanol and cyan for acetone); bottom: output of the PLS-DA classifier and mean sensor array response (for visual clarity, we added a threshold that indicates the mean array response during the commutation from air to ethanol).

Figure 10 shows the exploration results obtained with two gas sources (ethanol and acetone) placed in the corridor of the experimentation area with the HVAC switched off. The evaporation of ethanol and acetone started simultaneously when the mobile robot started the exploration. The center of mass of the area detected as ethanol or acetone appeared displaced from the gas sources, probably because the experiment was performed on a summer day at noon with the highest level of solar radiation. Therefore, this displacement was probably caused by the natural convection originating from the existing temperature gradient between the interior (26.5 °C in the corridor) and exterior (31.4 °C) of the building.

### 3.6. Scenario IV: Gas Leak Inside a Door-Closed Room and HVAC Turned On

**Figure 11:**
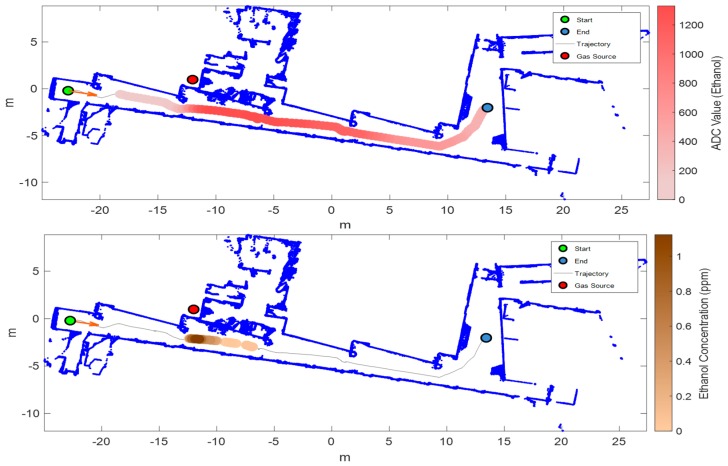
Results of exploration with an ethanol gas source inside a closed room and HVAC turned on (Scenario IV). Top: map of the arena (built by the robot) and mean sensor array response overlaid on the robot trajectory; bottom: map of the arena (built by the robot) and PID response overlaid on the robot trajectory.

Figure 11 shows the results of the exploration with a leak of ethanol located inside a laboratory with the door closed and the HVAC turned on. The mobile robot is exploring the corridor and does not enter the room. In this case, the gas spreads outside of the room under the door because of the global air recirculation originated by the HVAC. The maximum mean response of the sensor array was obtained near the door of the closed laboratory, although the gas sensor array was able to detect the ethanol a few meters before reaching that location. The PID provides a more accurate estimate of the source location but only detects the gas when the robot is in front of the door of the room containing the gas source. The results of this experiment demonstrate that a gas leak originated inside a closed laboratory can be detected by a mobile robot at a large distance from the source with an embedded array of MOX gas sensors when the HVAC of the building is turned on.

## 4. Conclusions and Future Work

This paper proposes the extension of the application of a mobile robot as an early gas leak detector for safety purposes. The gas sensor is composed of an array of 16 low-cost MOX sensors which are continuously powered for continuous measurement. The measurements were carried out with the mobile robot in continuous motion without stopping for taking measurements. The matrix of gas sensors generates 16 individual voltage measurements which are sampled with a 10-bit ADC and converted to sensor conductance. Additionally, all sensor voltage values sampled are added and used as a fast indicator of a change in the gas concentration by applying a threshold level. Then, the MOX sensor conductance is applied to a trained PLS-DA in order to classify the gas as air, ethanol, or acetone. Results show that the proposed system can detect two chemical sources in an indoor scenario at large distances from the source. The proposed classifier was able to correctly classify the target gas in a real scenario, despite the validation conditions being more challenging than the calibration conditions. Results also show that, when two sources are present at the same time, the mobile robot detects only the most concentrated chemical. The combination of mobile robot and gas sensing device was able to detect a gas leak located in a contiguous room with closed doors due to the small air flow going under the door. The positive results obtained in different operation conditions over the course of one month confirmed the early detection capabilities of the proposed mobile system.

## Figures and Tables

**Table 1:** Metal-oxide (MOX) sensors used in the electronic board and duty cycle applied. ID—identifier; PWM—pulse width modulation.

ID	Sensor	PWM Channel	Duty Cycle
1	TGS 2600	1	25%
2	TGS 2602	1	25%
3	TGS 2611	1	25%
4	TGS 2620	1	25%
5	TGS 2620	2	50%
6	TGS 2611	2	50%
7	TGS 2602	2	50%
8	TGS 2600	2	50%
9	TGS 2620	3	75%
10	TGS 2611	3	75%
11	TGS 2602	3	75%
12	TGS 2600	3	75%
13	TGS 2620	4	62.5%
14	TGS 2611	4	62.5%
15	TGS 2602	4	62.5%
16	TGS 2600	4	62.5%

## References

[1] Martinez D., Teixidó M., Font D., Moreno J., Tresanchez M., Marco S., Palacín J. (2014). Ambient intelligence application based on environmental measurements performed with an assistant mobile robot. Sensors.

[2] Palacín J., Clotet E., Martínez D., Moreno J., Tresanchez M. (2017). Automatic Supervision of Temperature, Humidity, and Luminance with an Assistant Personal Robot. J. Sens..

[3] Jin M., Liu S., Schiavon S., Spanos C. (2018). Automated mobile sensing: Towards high-granularity agile indoor environmental quality monitoring. Build. Environ..

[4] Reggente M., Mondini A., Ferri G., Mazzolait B., Manzi A., Gabelletti M., Dario P., Lilienthal A.J. (2010). The DustBot System: Using Mobile Robots to Monitor Pollution in Pedestrian Area. Chem. Eng. Trans..

[5] Soldan S., Bono G., Kroll A. (2012). RoboGasInspector—A Mobile Robotic System for Remote Leak Sensing and Localization in Large Industrial Environments: Overview and First Results. IFAC Proc. Vol..

[6] Hernandez V., Lilienthal A.J., Neumann P.P., Trincavelli M. (2012). Mobile robots for localizing gas emission sources on landfill sites: Is bio-inspiration the way to go?. Front. Neuroeng..

[7] Rossi M., Brunelli D., Adami A., Lorenzelli L., Menna F., Remondino F. Gas-Drone: Portable gas sensing system on UAVs for gas leakage localization. Proceedings of the 2014 IEEE SENSORS.

[8] Gallego V., Rossi M., Brunelli D. Unmanned aerial gas leakage localization and mapping using microdrones. Proceedings of the 2015 IEEE Sensors Applications Symposium.

[9] Rossi M., Brunelli D. (2016). Autonomous Gas Detection and Mapping with Unmanned Aerial Vehicles. IEEE Trans. Instrum. Meas..

[10] Kowadlo G., Russell R.A. (2008). Robot Odor Localization: A Taxonomy and Survey. Int. J. Robot. Res..

[11] Monroy J., Gonzalez-Jimenez J. (2018). Towards Odor-Sensitive Mobile Robots—Electronic Nose Technologies and Advances in Machine Olfaction. IGI Glob..

[12] Monroy J., Ruiz-Sarmiento J.-R., Moreno F.-A., Melendez-Fernandez F., Galindo C., Gonzalez-Jimenez J. (2018). A Semantic-Based Gas Source Localization with a Mobile Robot Combining Vision and Chemical Sensing. Sensors.

[13] Sanchez-Garrido C., Monroy J., Gonzalez-Jimenez J. (2018). Probabilistic Estimation of the Gas Source Location in Indoor Environments by Combining Gas and Wind Observations. Appl. Intell. Syst..

[14] Li J.-G., Meng Q.-H., Wang Y., Zeng M. (2011). Odor source localization using a mobile robot in outdoor airflow environments with a particle filter algorithm. Auton. Robots.

[15] Bennetts V.H., Schaffernicht E., Lilienthal A.J., Trincavelli M. Robot Assisted Gas Tomography—Localizing Methane Leaks in Outdoor Environments. Proceedings of the IEEE International Conference on Robotics and Automation.

[16] Lilienthal A.J., Duckett T. (2004). Building gas concentration gridmaps with a mobile robot. Robot. Auton. Syst..

[17] Loutfi A., Coradeschi S., Lilienthal A.J., Gonzalez J. (2009). Gas Distribution Mapping of Multiple Odour Sources using a Mobile Robot. Robotica.

[18] Hernandez V., Lilienthal A.J., Trincavelli M. Creating true gas concentration maps in presence of multiple heterogeneous gas sources. Proceedings of the Conference IEEE Sensors 2012.

[19] Burgués J., Hernández V., Lilienthal A.J., Marco S. (2019). Smelling Nano Aerial Vehicle for Gas Source Localization and Mapping. Sensors.

[20] Monroy J.G., Blanco J.L., Gonzalez-Jimenez J. (2016). Time-variant gas distribution mapping with obstacle information. Auton. Robots.

[21] Monroy J.G., Gonzalez-Jimenez J. (2017). Gas classification in motion: An experimental analysis. Sens. Actuators B Chem..

[22] Burgués J., Jiménez-Soto J.M., Marco S. (2018). Estimation of the limit of detection in semiconductor gas sensors through linearized calibration models. Anal. Chim. Acta.

[23] Burgués J., Marco S. (2018). Low Power Operation of Temperature-Modulated Metal Oxide Semiconductor Gas Sensors. Sensors.

[24] Wang C., Yin L., Zhang L., Xiang D., Gao R. (2010). Metal Oxide Gas Sensors: Sensitivity and Influencing Factors. Sensors.

[25] Fine G.F., Cavanagh L.M., Afonja A., Binions R. (2010). Metal Oxide Semi-Conductor Gas Sensors in Environmental Monitoring. Sensors.

[26] Jiu H.F., Pang S., Li J.L., Han B. Odor plume source localization with a Pioneer 3 Mobile Robot in an indoor airflow environment. Proceedings of the IEEE SOUTHEASTCON 2014.

[27] Bennetts V.H., Schaffernicht E., Pomareda V., Lilienthal A.J., Marco S., Trincavelli M. (2014). Combining non selective gas sensors on a mobile robot for identification and mapping of multiple chemical compounds. Sensors.

[28] Clotet E., Martínez D., Moreno J., Tresanchez M., Palacín J. (2016). Assistant Personal Robot (APR): Conception and Application of a Tele-Operated Assisted Living Robot. Sensors.

[29] Palacín J., Clotet E., Martínez D., Martínez D., Moreno J. (2019). Design, Extending the Application of an Assistant Personal Robot as a Walk-Helper Tool. Robotics.

[30] Sánchez-Sosa J.E., Castillo-Mixcóatl J., Beltrán-Pérez G., Muñoz-Aguirre S. (2018). An Application of the Gaussian Plume Model to Localization of an Indoor Gas Source with a Mobile Robot. Sensors.

[31] Moreno J., Clotet E., Lupiañez R., Tresanchez M., Martínez D., Pallejà T., Casanovas J., Palacín J. (2016). Design, Implementation and Validation of the Three-Wheel Holonomic Motion System of the Assistant Personal Robot (APR). Sensors.

[32] Moreno J., Clotet E., Tresanchez M., Martínez D., Casanovas J., Palacín J. (2017). Measurement of Vibrations in Two Tower-Typed Assistant Personal Robot Implementations with and without a Passive Suspension System. Sensors.

[33] Martínez D., Clotet E., Moreno J., Tresanchez M., Palacín J. (2016). A Proposal of a Multi-agent System Implementation for the Control of an Assistant Personal Robot. Advances in Intelligent Systems and Computing (AISC 473), Trends in Practical Applications of Scalable Multi-Agent Systems.

[34] Thrun S. (2002). Robotic mapping: A survey. Exploring Artificial Intelligence in the New Millennium.

[35] Boal J., Sánchez-Miralles Á., Arranz Á. (2014). Topological simultaneous localization and mapping: A survey. Robotica.

[36] Wang Y.-T., Peng C.-C., Ravankar A.A., Ravankar A. (2018). A Single LiDAR-Based Feature Fusion Indoor Localization Algorithm. Sensors.

[37] Hart P.E., Nilsson N.J., Raphael B. (1968). A formal basis for the heuristic determination of minimum cost paths. IEEE Trans. Syst. Sci. Cybern..

[38] Yang D., Xu B., Rao K., Sheng W. (2018). Passive Infrared (PIR)-Based Indoor Position Tracking for Smart Homes Using Accessibility Maps and A-Star Algorithm. Sensors.

[39] Martínez D., Moreno J., Tresanchez M., Clotet E., Jiménez-Soto J.M., Magrans R., Pardo A., Marco S., Palacín J. (2016). Measuring Gas Concentration and Wind Intensity in a Turbulent Wind Tunnel with a Mobile Robot. J. Sens..

[40] Pomareda V., Magrans R., Jiménez-Soto J.M., Martínez D., Tresánchez M., Burgués J., Palacín J., Marco S. (2017). Chemical Source Localization Fusing Concentration Information in the Presence of Chemical Background Noise. Sensors.

[41] Fonollosa J., Rodríguez-Luján I., Trincavelli M., Vergara A., Huerta R. (2014). Chemical Discrimination in Turbulent Gas Mixtures with MOX Sensors Validated by Gas Chromatography-Mass Spectrometry. Sensors.

[42] Madrolle S., Grangeat P., Jutten C. (2018). A Linear-Quadratic Model for the Quantification of a Mixture of Two Diluted Gases with a Single Metal Oxide Sensor. Sensors.

[43] Fonollosa J., Fernández L., Gutiérrez-Gálvez A., Huerta R., Marco S. (2016). Calibration transfer and drift counteraction in chemical sensor arrays using Direct Standardization. Sens. Actuators B Chem..

[44] Rossi M., Brunelli D. Ultra low power Wireless Gas Sensor Network for environmental monitoring applications. Proceedings of the IEEE Workshop on Environmental Energy and Structural Monitoring Systems (EESMS).

[45] Jeličić V., Magno M., Paci G., Brunelli D., Benini L. Design, characterization and management of a wireless sensor network for smart gas monitoring. Proceedings of the 2011 4th IEEE International Workshop on Advances in Sensors and Interfaces (IWASI).

[46] Rossi M., Brunelli D. Analyzing the transient response of MOX gas sensors to improve the lifetime of distributed sensing systems. Proceedings of the 5th IEEE International Workshop on Advances in Sensors and Interfaces IWASI.

[47] Brunelli D., Rossi M. (2014). Enhancing lifetime of WSN for natural gas leakages detection. Microelectron. J..

[48] Ballabio D., Consonni V. (2013). Classification tools in chemistry. Part 1: Linear models. PLS-DA. Anal. Methods.

[49] Burgués J., Marco S. (2018). Multivariate estimation of the limit of detection by orthogonal partial least squares in temperature-modulated MOX sensors. Anal. Chim. Acta.

